# Multiple Weather Factors Affect Apparent Survival of European Passerine Birds

**DOI:** 10.1371/journal.pone.0059110

**Published:** 2013-04-08

**Authors:** Volker Salewski, Wesley M. Hochachka, Wolfgang Fiedler

**Affiliations:** 1 Department of Migration and Immuno-ecology, Max-Planck-Institute for Ornithology, Radolfzell, Germany; 2 Lab of Ornithology, Cornell University, Ithaca, New York, United States of America; Institute of Ecology, Germany

## Abstract

Weather affects the demography of animals and thus climate change will cause local changes in demographic rates. In birds numerous studies have correlated demographic factors with weather but few of those examined variation in the impacts of weather in different seasons and, in the case of migrants, in different regions. Using capture-recapture models we correlated weather with apparent survival of seven passerine bird species with different migration strategies to assess the importance of selected facets of weather throughout the year on apparent survival. Contrary to our expectations weather experienced during the breeding season did not affect apparent survival of the target species. However, measures for winter severity were associated with apparent survival of a resident species, two short-distance/partial migrants and a long-distance migrant. Apparent survival of two short distance migrants as well as two long-distance migrants was further correlated with conditions experienced during the non-breeding season in Spain. Conditions in Africa had statistically significant but relatively minor effects on the apparent survival of the two long-distance migrants but also of a presumably short-distance migrant and a short-distance/partial migrant. In general several weather effects independently explained similar amounts of variation in apparent survival for the majority of species and single factors explained only relatively low amounts of temporal variation of apparent survival. Although the directions of the effects on apparent survival mostly met our expectations and there are clear predictions for effects of future climate we caution against simple extrapolations of present conditions to predict future population dynamics. Not only did weather explains limited amounts of variation in apparent survival, but future demographics will likely be affected by changing interspecific interactions, opposing effects of weather in different seasons, and the potential for phenotypic and microevolutionary adaptations.

## Introduction

Weather has important direct and indirect effects on the demography of organisms [1]. Thus changes in local weather through climate change will proximally affect demographic rates, and ultimately affect future global biodiversity and species assemblages [2], [3]. Therefore, identifying the effects of weather on demography is important for anticipating the effects of climate change. Seasonality complicates this process because influences of weather may differ among seasons, with survival or reproduction affected directly or indirectly through carry-over effects in which the consequences of non-lethal conditions in one season become manifested in another season [4]. For migratory animals, identification of key weather influences on demography requires examination of potentially important facets of weather in different times and regions, including effects that carry over from one season and location to the next [5].

To date, examinations of the influences of weather on population dynamics of birds have rarely considered these complexities, instead focussing on one or a small number of potential critical time periods or geographic locations rather than looking at the annual cycle as a whole. An example are the contrasting “tub” and “tap” hypotheses [6]: the “tub” hypothesis is that fluctuations in population size are associated with weather variation in the non-breeding season, and the “tap” hypothesis that fluctuations depend on the weather during the breeding season. Sæther et al. [6] found that in general the tub hypothesis was supported in northern temperate altricial birds whereas the tap hypothesis explained population fluctuations of many nidifugous species and of those living under arid conditions. However, these generalizations may obscure more complex patterns, as studies have shown that: conditions during the non-breeding season can have differing influences on geographically separated populations of the same species [7]; individual survival, but not population dynamics is correlated with conditions in the non-breeding season [8]; and conditions in both non-breeding season and breeding season influence individual survival of migrating birds [9], [10].

Birds breeding in Central Europe experience fluctuating climatic conditions in seasonal environments throughout the annual cycle whether they are residents or migrants. While the demography of resident species is influenced by fluctuating conditions in only one region, migratory species experience influences of local weather at multiple locations. These migrants typically travel along a south-westerly route from their nesting grounds to southern wintering areas either in the western Mediterranean (short-distance migrants) or in the savanna zones of sub-Saharan West Africa (long-distance migrants). Current predictions for climate change during the breeding season of birds suggest warmer and drier conditions in Central Europe [11], [12]. Predictions for the non-breeding season of resident or migrating Central European bird species suggest increasing winter temperatures and precipitation will be encountered in Central Europe [12], [13] and increasing temperatures as well as decreasing precipitation in the western Mediterranean [12]. The non-breeding areas of long-distance migrants to West Africa are predicted to experience a general increase in temperature, but a decrease in precipitation of 10% to >20% by the end of this century [14]. Given these scenarios, the predicted changes in climate may improve future breeding conditions for many species (but see [15]). Furthermore, future conditions during the non-breeding season in Europe may be advantageous for resident birds. In contrast the predicted changes in West Africa during the non-breeding season may be disadvantageous to migrants' survival during the non-breeding period [16]–[18] as well as for reproduction in Europe because of negative carry-over effects [19], [20]. Given the contrasting predictions for climate change across the regions occupied by Central European birds throughout the year, any attempts to anticipate the impacts of climate change will require identification of the weather conditions and geographic locations for which variation in weather most influences population dynamics.

The aim of this study is to assess the relative importance of the variation of multiple facets of weather, recorded at locations at which birds breeding in Central Europe reside throughout the year. We relate these measures of weather to apparent survival of seven passerine bird species that have contrasting migration strategies, although all breed in the same geographic area (south-western Germany). This allows us to determine whether the same facets of weather are correlated with apparent survival of species with differing lengths of migratory travel and whether single weather factors can explain a high variation of apparent survival. We first identify which of a set of weather factors affect the bird species' apparent survival, specifically looking for evidence supporting three non-exclusive hypotheses: (1) all species have apparent survival rates that are affected by weather conditions on their breeding grounds; (2) both short- and long-distance migrants are affected by weather conditions in the Mediterranean and (3) apparent survival of long-distance migrants is affected by weather conditions in their sub-Saharan wintering grounds. Second, we determine whether any single weather feature explains a high variation in apparent survival compared to other factors. Finally we assess the implications of the identified impacts of weather variation for future population dynamics given the current prediction for the ongoing climate change.

## Methods

### Ethic statement

All handling of birds was carried out in strict compliance with the laws of Germany and the European Union. Trapping and banding birds for scientific purposes in Germany is subject of the species conservation legislation (§§ 38, 39 Bundesnaturschutzgesetz) and does not fall under the animal welfare legislation in the sense of an animal experiment (§ 7 Tierschutzgesetz). Therefore under German law approval of the study by an ethics committee is not required. All studies involving trapping and banding of birds are evaluated by the Bird Ringing Centre and the institutional Animal Welfare Officer or his deputy. Guidelines for the responsible handling of captured wild birds (issued by the Bird Ringing Centre) apply and all persons handling birds received training and supervision by licensed senior bird banders. The permit to trap and band birds within the framework of this study was issued by the responsible authority Regierungspräsidium Freiburg under the No. 56-8853.17/01.

### Study area and target species

Birds were captured as part of a monitoring programme on the Mettnau peninsula (47.729°N, 8.998°E), Lake Constance, near Radolfzell in south-western Germany. Starting in 1972, and between 30 June to 6 November inclusive, birds were captured in mist-nets following standardised methods [21]. The early start of the netting season enabled the capture of local breeding birds as well as migrating individuals of most species. All captured birds were identified to species, banded and standard measurements were taken. Captured birds were handled within one hour and immediately released after measurements were taken. All recaptures were also recorded with at least date of recapture and band number noted. Birds were aged following the criteria in Svensson [22] and Jenni & Winkler [23] as being in their first year (i.e. captured in their first year of life) or older. For this study we analysed data from bird captures in the years 1972 (1973 in blackbirds *Turdus merula*) through 2005, inclusive.

We went through several steps in order to select the species and ranges of capture dates for each species used in our analyses. We included only individuals that were in their first year of life when captured for the first time in order to consider only capture histories that potentially cover entire life spans. Our basic criterion for including a species in our analysis was that at least 1,000 first-year individuals were captured. Our second criterion was the presence of a relatively high inter-annual recapture rate; therefore we selected only species for which we had ≥2.5% inter-annual recaptures. Following Salewski et al. [24] we identified dates on which netted individuals of a species were likely to have originated from the immediate region and excluded putative non-regional migrating birds from our analyses.

The seven species in our final set (Table 1) have a range of patterns of movement between summer and winter. In blackbirds the majority of first year birds are probably short-distance migrants whereas there is an increasing trend to be resident in older blackbirds especially in males [25]. *Dunnocks Prunella* modularis are probably partial migrants with an unknown proportion migrating to areas as distant as the Mediterranean, while others winter much closer to the breeding grounds [26]. Blackcaps *Sylvia atricapilla* and reed buntings *Emberiza schoeniclus* are short distance migrants to the Mediterranean region [26], but an increasing number of blackcaps spend the non-breeding season in Britain and Ireland [27], [28]. The great majority of chiffchaffs *Phylloscopus collybita* from the study region are probably also short-distance migrants to the Mediterranean [29]. Whether the increasing numbers of winter observations in the region [30] are of local breeders is unknown. Reed warblers *Acrocephalus scirpaceus* and willow warblers *Phylloscopus trochilus* are long-distance migrants to tropical Africa and band recoveries indicate that birds from western and central Europe spent the European winter months in the West African Guinea savanna zone south of 10°N [29], [31].

**Table 1:** Target species.

Species	n	% recaptured	Date after newly-captured considered to be non-local
Dunnock *Prunella modularis*	596	8.2	6 September
Blackbird *Turdus merula*	1178	4.5	25 September
Reed Warbler *Acrocephalus scirpaceus*	1879	6.3	13 July
Blackcap *Sylvia atricapilla*	4636	2.5	22 August
Chiffchaff *Phylloscopus collybita*	9482	2.9	22 September
Willow Warbler *Phylloscopus trochilus*	1734	7.6	22 July
Reed Bunting *Emberiza schoeniclus*	4386	7.1	13 September

Shown are the number of first year birds captured per species, percentage recaptures in a subsequent year and date after which an individual of the respective species was classified as a migrant and not considered in the analysis.

Although we did not consider an explicit breeding population, (i.e. birds marked in or at their nest), we considered only individuals that were initially banded within a short time after presumed fledging. Although we may have included juveniles performing premigratory dispersal movements apparent survival rates of first year individuals of most species and adult individuals of all species were comparable to those found in literature (unpubl. data). Therefore we are convinced that we have mainly considered locally raised birds.

### Apparent survival and the selection of weather variables

While our goal was to identify weather factors that affect survival of birds, we can only estimate apparent survival (Ф), which is [32] a function of true survival (S) and the probability of permanent emigration (d):
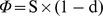



While a very large number of weather variables can be obtained or derived, we deliberately selected a small number to examine because: prior studies have already identified candidates, restricting the set of predictors that is examined will lower the probability of finding spurious correlations, and many variables describing weather are correlated and we did not want to include redundant predictors in the set that we examined. Whilst the relevance of any specific weather variables for prediction of survival can be debated, we describe the rationale for our choices in detail below. Most of the weather variables that we examine have been previously used, albeit as subsets of our entire set in any individual study, thus facilitating comparisons with previous studies.

Based on prior studies, we tested whether local precipitation (details below) during the breeding season as the feature of weather that potentially affects apparent survival. High local precipitation during the breeding season is typically associated with low breeding success in Central Europe [33]–[35]. Additionally, precipitation may affect dispersal, with breeding dispersal (dispersal from one breeding site to another) becoming lower with higher breeding success [20], [36], [37]. In general first year birds have a lower rate of philopatry to a breeding site than older birds [36]: young birds have to disperse to breed elsewhere with higher philopatry of older birds. Therefore, we expected that higher precipitation during the breeding season would be associated with lower apparent survival for older birds but relatively increased apparent survival of first year birds.

Weather in the non-breeding season can have profound effects on birds' survival [6] and low winter temperatures as well as snow cover may be associated with increased winter mortality and decreasing population sizes either because of thermoregulatory challenges or reduced food supply [38]–[40]. Therefore we selected winter temperatures and days with snow cover as potential predictors of apparent survival due to their effects on winter survival for resident species, and we expected higher survival of these resident species with warmer winter temperatures and less snow. Prior work suggests that rainfall in wintering areas is a predictor of apparent survival for migrants [8], [16], [17], and therefore we included in our models precipitation in Spain as well as in the African Sahel and Guinea savanna zones. We assumed a priori that dry conditions during winter in temperate Spain would be advantageous for insectivorous birds because their prey would be more active although we are not aware of any studies confirming our assumption explicitly. Although the Sahel zone is not the final non-breeding area of the two long-distance migrants considered here, at least willow warblers may stay there for a period of time after crossing the Sahara in autumn [41]. The African savanna zones may also play a role in premigratory fattening in spring for both species. Therefore we expected a higher survival with greater rainfall in Africa because increased precipitation is associated with higher primary production and insect abundance in African savannas [42]–[44].

Data on precipitation during the breeding season are standard daily measurements taken at Konstanz (47,667°N, 9,183°E) by the Deutscher Wetterdienst, approximately 18 km from the study site (http://www.dwd.de/de/FundE/Klima/KLIS/daten/online/nat/index_standardformat.htm). From the sum of the daily values we calculated the mean daily precipitation for the period from 1 April to 30 June for each year. Local winter temperatures and days with snow cover were extracted from the same source. Daily mean temperatures were used to calculate the mean temperature between 1 December and the last day of February for each winter. For snow cover we used the sum of days with a closed snow cover (snow height ≥1 cm) between 1^st^ October and 31^st^ March as a second surrogate for winter severity. Extreme weather events or periods of adverse conditions may be more strongly correlated with apparent survival and population dynamics than multi-day averages [40], [45], [46]. However, in our study area extremes and averages were correlated: mean daily winter temperature was significantly negatively associated with the number of consecutive days with a mean temperature below 0°C (linear model: df  = 31, adjusted r^2^  = 0.20, p = 0.005) and the mean number of days with snow cover per winter was significantly positively correlated with the maximum number of consecutive days with snow cover per winter (linear model: df  = 31, adjusted r^2^  = 0.65, p = <0.001). Therefore, while we are using mean values as predictors in our analyses, interpretation of results needs to be made knowing that longer-term means as well as shorter-term extremes were described by our chosen measures of winter weather.

The Sahel precipitation-index was taken from the website of the Joint Institute for the Study of the Atmosphere and Ocean (http://jisao.washington.edu/data/sahel/). No such precipitation indices covering the entire study period are available from the western Mediterranean and the Guinea Savanna-zone. Therefore we calculated precipitation indices from data from individual weather stations taken from the CISL Research Data Archive (http://dss.ucar.edu/). For Spain we used precipitation data from five sites between 37.4°N and 41.7°N and between 6.1°W and 2.1°E (Alicante, Barcelona, Madrid, Seville, Valladolid). For the Guinea-index we used precipitation data for seven localities between 6.2°N and 8.0°N and between 10.4°W and 2.4°E. The latter were Roberts Field in Liberia; Atakpame, Lomé and Tabligbo in Togo and Bohincon, Cotonou and Savé in Benin. We used monthly data from November to February, inclusive, which is the main staging period of migrants in the respective regions. Data for a number of months were missing from a number of weather stations. We therefore needed to calculate an index of winter weather that interpolated over these missing data rather than calculating simple averages from only the available data. We used the parameter estimates for “winter” from an ANOVA with “precipitation” (monthly value) as the dependent variable and “winter”, “month” and “site” as the independent variable to correct precipitation data for site- and month-specific effects.

Large scale climate indices may predict ecological processes better than local weather data [47]. Additionally, for partial migrants (dunnock, blackbird) or for populations in which individuals migrate to different destinations (blackcap) a large scale index may describe apparent survival more adequately than a local factor that affects only a part of the population. Therefore, we also included the North Atlantic Oscillation Index (NAO) during the winter months (December – March, [48]) in our set of factors potentially affecting apparent survival. A low NAO is associated with relatively colder drier winters and a higher index is associated with warmer, wetter winters in northern Europe. In the Mediterranean a low index is associated with drier winters [49]. Therefore we predicted a higher apparent survival for resident birds and short distance migrants in association with a higher NAO index. As expected the NAO was significantly positively associated with the winter temperature (linear model: df  = 31, adjusted r^2^  = 0.12, p = 0.026) and negatively associated with snow cover (linear model: df  = 31, adjusted r^2^  = 0.09, p = 0.050) in the breeding area. The association of the NAO with the other weather variables were not statistically significant. The data for the NAO were downloaded from NASA's Global Change Master Directory (http://gcmd.nasa.gov/KeywordSearch/Metadata.do?Portal=GCMD&KeywordPath=&NumericId=4689&MetadataView=Full&MetadataType=0&lbnode=mdlb2).

Weather data from the breeding area were z-transformed prior to the analyses, but not the indices from the migration and wintering areas and the NAO. In our models the factors are denoted as follows: breeding season precipitation  =  bsp, local winter temperature  =  wt, local snow cover  =  sn, winter precipitation in Spain  =  pSp, precipitation in the African Sahel zone  =  pSz and precipitation in the Guinea savanna zone  =  pGz.

While we have specific expectations for how the selected facets of weather will have affected the birds that are wintering in the regions in which weather is described, weather effects may be more complex, with conditions in one time period in a region affecting population dynamics of bird populations although they are not present in the region at that particular time. For example, the population size for wood warblers *Phylloscopus sibilatrix*, a species that migrates to tropical Africa in the non-breeding season, shows declines correlated with severe winters in Europe [50]. Because of the potential for these complex inter-relationships, we looked for evidence of relationships between all variables with apparent survival of all species irrespective of their non-breeding season distributions.

### Data analyses

It is important to test whether the mark-recapture models that we used were adequate descriptions of the processes that we want to describe. For this purpose we performed goodness-of-fit (GOF) tests using the program U-care [51]. The accepted significance level was p ≤0.05. The test 3.SR for transient and age effects was significant for all species except for the reed warbler (Table 2). Therefore we incorporated age structuring (a_2_) in all our models. A goodness of fit test when all first captures were removed was never significant for either U-care's global test or for any of the specific tests, indicating that there is no over dispersion in the data. Therefore we did not correct our output statistics with a ĉ – value.

**Table 2:** Probabilities from goodness-of-fit tests.

Species	Statistic	Test				
		Global	3.SR	3.SM	2.CT	2.CL
Dunnock	df	37	20	7	8	2
	χ^2^	34.611	32.031	0.000	1.644	0.936
	p	0.582	0.043	1.000	0.990	0.626
Blackbird	df	54	25	7	16	6
	χ^2^	54.848	42.870	0.096	7.537	3.516
	p	0.442	0.014	0.996	0.961	0.742
Reed Warbler	df	55	26	8	15	6
	χ^2^	25.902	11.283	0.936	12.975	0.708
	p	1.000	0.995	0.999	0.604	0.994
Blackcap	df	73	29	11	21	12
	χ^2^	57.163	44.114	3.490	5.917	3.641
	p	0.914	0.036	0.983	0.999	0.989
Chiffchaff	df	63	27	12	18	6
	χ^2^	69.501	45.083	8.450	14.324	1.644
	p	0.268	0.016	0.749	0.708	0.949
Willow Warbler	df	45	26	10	8	1
	χ^2^	62.644	51.743	3.540	6.425	0.936
	p	0.042	0.002	0.966	0.600	0.333
Reed Bunting	df	97	30	21	27	19
	χ^2^	116.547	78.848	6.858	22.668	8.174
	p	0.086	<0.001	0.998	0.703	0.985

Shown are the degrees of freedom, χ^2^ and p-values for the global test as well as for the tests 3.SR, 3.SM, 2.CT and 2.CL.

We used the program MARK 4.2 [52] to estimate rates of apparent survival (Φ) with mark-recapture analyses. We performed a three step analysis to limit the number of candidate models that we examined [53], [54]. Capture probabilities (p) are nuisance factors in our study, but inappropriate models of capture probabilities will bias apparent survival estimates of the model. Thus, our initial step was to determine the appropriate model descriptions of recapture probability, comparing models in which parameters for apparent survival were modelled as having an age structure and were time dependent and treated recapture probability as either: varying among years but with no linear trend, with a linear time trend or as invariant through time. Following Lebreton et al. [53] these models are denoted as {Φ(a_2_*t) p(t)}, {Φ(a_2_*t) p(T)} and {Φ(a_2_*t) p(.)} respectively. The most parsimonious recapture model was the model {Φ(a_2_*t) p(.)} for all species except for the willow warbler for which the most parsimonious model was {Φ(a_2_*t) p(T)}, with a strong linear decline in between-year recapture probability throughout the study period. Therefore, except for the willow warbler, we kept the recapture probability constant in the second step of our analyses to model apparent survival probabilities using the logit link function in MARK. For the willow warbler we included a linear time trend for the recapture probability in all models. This second step compared a set of models, in which age (first year or older) was modelled as an interaction with either a single weather factor, time either as a categorical variable (t), or time as a linear trend (T), as well as a model in which age alone was a predictor. In a third step we then rerun the most parsimonious model from step two with the three capture probabilities that we examined in the first step, but this third step did not lead to a different model being selected. Therefore we do only present the results of the analyses after step two. The final set of models is found in Table 3. Akaike's Information Criterion for small sample sizes (AIC_C_, [55]) was used to rank the models and the model with the lowest AIC_C_-value is the most parsimonious one that is best supported by the data. All models with a ΔAIC_C_ ≤2 are treated as having similar support from the data compared to the most parsimonious model [55].

**Table 3:** Apparent survival in relation to climate variables, calculated using capture-mark-recapture analyses, for seven passerine bird species.

Model	Species													
	Dunnock		Blackbird		Reed warbler		Blackcap		Chiffchaff		Willow warbler		Reed bunting	
	ΔAIC_C_	AIC_C_W	ΔAIC_C_	AIC_C_W	ΔAIC_C_	AIC_C_W	ΔAIC_C_	AIC_C_W	ΔAIC_C_	AIC_C_W	ΔAIC_C_	AIC_C_W	ΔAIC_C_	AIC_C_W
Φ(a_2_)	1.2	0.153	5.6	0.035	2.1	0.113	3.6	0.084	13.7	0.001	4.5	0.034	9.8	0.007
Φ(a_2*bsp_)	3.1	0.060	2.8	0.140	5.2	0.023	6.7	0.017	11.9	0.002	2.3	0.099	10.6	0.005
Φ(a_2*wt_)	0.1	0.260	0.0^1^	0.556^1^	4.6	0.031	5.2	0.038	7.0	0.029	4.5	0.034	13.0	0.001
Φ(a_2*sn_)	4.8	0.025	2.0	0.204	3.2	0.062	4.5	0.052	0.0^1^	0.958^1^	0.0^1^	0.319^1^	11.5	0.003
Φ(a_2*pSp_)	3.7	0.045	9.5	0.005	0.6	0.239	2.0	0.187	16.3	<0.001	1.4	0.159	0.0^1^	0.962^1^
Φ(a_2*pSz_)	1.8	0.116	6.8	0.019	1.4	0.159	6.9	0.016	14.8	0.001	1.7	0.137	9.4	0.009
Φ(a_2*pGz_)	4.3	0.032	8.5	0.008	4.3	0.036	0.0^1^	0.506^1^	9.6	0.008	4.9	0.028	9.3	0.009
Φ(a_2*NAO_)	0.0^1^	0.280^1^	9.3	0.005	0.0^1^	0.316^1^	3.7	0.081	16.2	0.003	4.3	0.038	13.5	0.001
Φ(a_2*T_)	4.5	0.030	5.9	0.029	5.6	0.019	6.6	0.012	14.8	0.001	1.5	0.153	12.4	0.002
Φ(a_2*t_)	80.1	<0.001	85.7	0.029	56.0	<0.001	57.9	<0.001	19.8	<0.001	34.1	<0.001	49.6	<0.001

Shown are the parameters included in each of the models that we used to predict apparent survival Φ, the ΔAIC values for small sample sizes (ΔAIC_C_) and the AIC weights (AIC_C_W) for all models. Recapture probability was always held constant {p(.), see methods} except for the willow warbler where it was modelled to have a linear effect {p(T)}. See methods for abbreviations of climate variables. ^1^: the most parsimonious model for a species.

Mortality of birds is high during their first months of life [56], [57] and natal dispersal is in general greater than breeding dispersal [58]. As both factors reduce the probability of apparent survival we assumed that apparent survival probability is lower for first year birds than for birds that have returned to the study site at least once. Apparent survival of first year birds is probably to a large extent explained by natal dispersal, but as birds tend to stay at the same breeding area once dispersed from the natal site [58] we assume that apparent survival of adult birds reflects mostly true survival, at least after breeding seasons with favourable breeding conditions.

In order to quantify the effect of the weather variables and the NAO on between-year apparent survival we calculated the proportion of deviance explained by these factors. According to Grosbois et al. [59] the proportion of deviance of the temporal variance explained by a model including a weather factor or the NAO is:
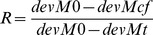



where devMt and devM0 are the deviances of the models {Φ(a_2*_t)p(.)} and {Φ(a_2_)p(.)} ({Φ(a_2*_t)p(T)} and {Φ(a_2_)p(T)} for the willow warbler). The deviance for the respective models including the weather factor or the NAO is devMcf.

## Results

### Importance of weather in different seasons and regions for apparent survival

Winter weather on the breeding grounds affected apparent survival of species with varying migration strategies, but rates of apparent survival were not influenced by precipitation during the breeding season for any species. This result (Table 3) was contrary to our first hypothesis. Winter temperature at the study site was included in the most parsimonious model for the resident blackbird (AIC_C_ weight  = 0.556) and had high support for the short distance migrant dunnock (AIC_C_ weight  = 0.260, Table 3). Snow cover was included in the most parsimonious models in the long-distance migrant willow warbler (AIC_C_ weight  = 0.319) and the short-distance migrant chiffchaff (AIC_C_ weight  = 0.958) and had high support for the resident blackbird (AIC_C_ weight  = 0.204, Table 3).

Our second hypothesis, that weather in the Mediterranean is an important predictor of apparent survival of both short- and long-distance migrants, was supported for four out of six migratory or partially migratory species (Table 3, but not the blackbird for which a high proportion of the population is probably resident). Precipitation in Spain was included in the most parsimonious model for the short-distance migrant reed bunting (AIC_C_ weight  = 0.962) and had high support for short-distance migrant blackcap (AIC_C_  = 0.187) and for the long-distance migrants reed warbler (AIC_C_ weight  = 0.239) and willow warbler (AIC_C_  = 0.159). Furthermore, the NAO was included in the most parsimonious model for the short distance migrant dunnock (AIC_C_ weight  = 0.280) and the long-distance migrant reed warbler (AIC_C_  = 0.316).

Our third hypothesis was also confirmed. The Sahel-index was included in the set of most parsimonious models (AIC_C_ ≤2) in the long-distance migrants reed warbler (AIC_C_  = 0.159) and willow warbler (AIC_C_ weight  = 0.137, Table 3). However, the model including the Sahel-index was also amongst the most parsimonious models in the short-distance migrant dunnock (AIC_C_  = 0.116) and the Guinea-index was included in the most parsimonious model in the short-distance migrant blackcap (0.506).

### Effects of weather variables on apparent survival

When weather features were identified as being important predictors of apparent survival (the most parsimonious models and those with a ΔAIC ≤2), our a priori expectations about the directions of association of age and of the weather factors were met in the majority of cases (Table 4). However, estimated magnitudes of weather effects were often small and standard errors relatively large. We expected apparent survival of first year birds to be lower than for birds that had already bred at the study site. This was the case in all of the sixteen models that had the highest support from the data. With respect to the weather factors, higher winter temperature (n = 4 species-age combinations, Table 4) in the breeding area was almost always associated with higher apparent survival. Only in first year dunnocks was a higher winter temperature associated with lower apparent survival. An increasing number of days with snow cover was always associated with lower apparent survival (n = 6 species-age combinations). The NAO was associated with higher apparent survival for both age classes of the reed warbler as well as in adult dunnocks but associated with lower apparent survival of first year dunnocks. Apparent survival was lower with increased precipitation during winter in Spain in both age classes of the reed warbler and the reed bunting, consistent with our expectation. However, in adult blackcaps and willow warblers of both age classes apparent survival was higher with increased precipitation in Spain. For the willow warbler we also found a general linear increase in apparent survival throughout the study period for both first year and adult birds (Table 4). With respect to precipitation on the wintering grounds of long-distance migrants, precipitation in the Sahel zone was positively correlated with apparent survival for both age classes of the reed warblers but only for adult willow warblers. Contrary to our expectation higher precipitation in the Guinea zone was associated with lower apparent survival of the blackcap. However, the support for this model for the latter species as well as the inclusion of the model including Sahel precipitation in the dunnock may be an artefact because this association makes little biological sense.

**Table 4:** ß-values (± se) of the models that included a weather variable with the highest support from the data.

	Dunnock	Blackbird	Reed Warbler	Blackcap	Chiffchaff	Willow Warbler	Reed Bunting
Model 1	Age_NAO_: −1.370±0.393^1^	Age: −2.713±0.343^1^	Age_NAO_: −0.788±0.383^1^	Age: −2.275±0.312^1^	Age: −1.433±0.216^1^	Age: −1.374±0.225^1^	Age: −1.374±0.169^1^
	NAO_fy_: −0.171±0.085	wt_fy_: 0.432±0.168^1^	NAO_fy_: 0.137±0.062^1^	pGz_fy_: −0.028±0.011	sn_fy_: −0.255±0.070^1^	sn_fy_: −0.154±0.099^1^	pSp_fy_: −0.011±0.003^1^
	NAO_ad_: 0.163±0.130^1^	wt_ad_: 0.430±0.318^1^	NAO_ad_: 0.068±0.091^1^	pGz_ad_: −0.029±0.020	sn_ad_: −0.247±0.158^1^	sn_ad_: −0.413±0.180^1^	pSp_ad_: −0.007±0.005^1^
Model 2	Age: −1.716±0.353^1^	Age: −2.667±0.345^1^	Age: −0.673±0.374^1^	Age: −2.173±0.297^1^		Age: −1.496±0.226^1^	
	wt_fy_: −0.304±0.171	sn_fy_: −0.131±0.672^1^	pSp_fy_: −0.009±0.005^1^	pSp_fy_: −0.009±0.004^1^		pSp_fy_: 0.011±0.004	
	wt_ad_: 0.355±0.258^1^	sn_ad_: −0.839±0.360^1^	pSp_ad_: −0.013±0.009^1^	pSp_ad_: 0.014±0.010		pSp_ad_: 0.003±0.008	
Model 3	Age: −2.537±0.576^1^		Age: −0.493±0.453^1^			Age: −1.610±0.402^1^	
	pSz_fy_: −0.002±0.005		pSz_fy_: 0.002±0.003^1^			T_fy_: 0.037±<0.001	
	pSz_ad_: 0.003±0.002^1^		pSz_ad_: 0.001±0.001^1^			T_ad_: 0.017±0.016	
Model 4						Age: −1.942±0.313^1^	
						pSz_fy_: −0.002±0.003	
						pSz_ad_: 0.002±0.001^1^	

Shown are the ß-values for the factor age (negative values indicate a lower apparent survival probability of first year birds) and for the climate variable for both age classes (fy: first year birds, ad: adult birds) for the most parsimonious model (Model 1) of the species and for the models with a ΔAIC_C_ ≤2.0 (Models 2–4). The models are indicated by the variables that are used to estimate apparent survival. ^1^: regression coefficients whose signs are consistent with a priori expectations. Note that we had no a priori expectations for the sign of the coefficient for the model including a constant trend in survival probability for the willow warbler.

### Single weather factors do not explain a large amount of variation of apparent survival

For only two species did a single model containing a weather variable have high support, as evidence by one or more additional model with support (ΔAIC_C_ >2, Table 3). The two species were the chiffchaff and the reed bunting, for which the model including snow cover on the breeding grounds and winter precipitation in Spain, respectively, had a higher support from the data than any other model. In the blackbird, however, the two models describing winter harshness (winter temperature, ΔAIC_C_  = 0.0, and snow cover, ΔAIC_C_  = 2.0) both explained a similar amount of apparent survival. Note, however, that in the dunnock models included variables (NAO, winter temperature) that were correlated with each other and some of the information in the model set may therefore be redundant.

None of the variables included in the most parsimonious models explained overwhelming proportions of the temporal variance of apparent survival of the target species (Table 5). The highest values were reached in the blackbird, the reed bunting and the chiffchaff where the models containing winter temperature, precipitation in Spain and snow cover explained 19.5%, 15.7% and 14.7% of the temporal variance of apparent survival, respectively. Additionally the model containing snow cover explained 15.5% of the temporal variance of apparent survival in the blackbird. In all other species no model explained much more than 10% of the temporal variance of apparent survival. Relatively high values were reached in the blackcap for which the model containing the Guinea-zone precipitation index explained 10.3% of apparent survival. A relatively high amount of variation in apparent survival was explained by the models containing the NAO in the dunnock (8.5%) and the reed warbler (7.9%) as well as by the models containing winter temperature for the dunnock (8.3%), precipitation in Spain for the blackcap (7.6%) and the reed warbler (7.2%) and snow cover for the willow warbler (8.2%).

**Table 5:** Proportions of the temporal variance that can be explained by each of the climate factors.

Species	Φ(NAO)	Φ(wt)	Φ(sn)	Φ(bsp)	Φ(pSp)	Φ(pSz)	Φ(pGz)
Dunnock	0.085^1^	0.083^1^	0.007	0.035	0.026	0.056^1^	0.015
Blackbird	0.006	0.195^1^	0.155^1^	0.139	0.001	0.057	0.021
Reed Warbler	0.079^1^	0.019	0.037	0.012	0.072^1^	0.061^1^	0.023
Blackcap	0.054	0.033	0.042	0.012	0.076^1^	0.010	0.103^1^
Chiffchaff	0.012	0.089	0.147^1^	0.048	0.012	0.024	0.067
Willow Warbler	0.041	0.039	0.082^1^	0.060	0.069^1^	0.066^1^	0.035
Reed Bunting	0.003	0.009	0.026	0.037	0.157^1^	0.050	0.051

Shown is the R^2^_dev_ for each factor for apparent survival Φ. ^1^: climate variables included in the most supported models (ΔAIC_C_ ≤2).

## Discussion

We found evidence that some facets of weather generally affected the apparent survival of the bird species that we examined: models containing measures of weather were in general better supported than models treating apparent survival as varying non-systematically over time or changing linearly through time. We showed that diverse facets of weather were associated with apparent survival. Nevertheless, our a priori hypotheses of specific effects of weather were not always met. Given the wide range of potential causes of mortality of wild birds throughout a year, we found that variation in our weather variables was not a dominant cause of inter-annual variation in apparent survival. Support of any model by the data was in general relatively low indicating that factors other than those considered by us are associated with apparent survival.

Contrary to our first hypothesis we found no support for effects of breeding-season weather on between-year apparent survival. Our results suggest that between-year apparent survival may depend more on weather affecting demography during the non-breeding season as in previous studies [10], [40], [60], [61]. Overall, these results support the contention that population dynamics of birds may be more affected by conditions during the non-breeding season [9], [62]–[64]. However, we have no data on breeding success and no data on the influence of weather on the number of recruits into breeding populations. Therefore our approach cannot directly test whether the tub-hypothesis (winter weather affects population size) or the tap-hypothesis (summer weather affects population size) explains fluctuations of populations.

With respect to our second hypothesis apparent survival of most migrants was affected by weather conditions in the western Mediterranean. To our knowledge this is the first study that found a relationship between precipitation in the western Mediterranean and apparent survival of Central European migrating passerines, and indicates that conditions during the non-breeding season can be associated with apparent survival of short-distance as well as long-distance migrants.

Confirming our third hypothesis, the model including Sahel-zone precipitation was amongst the most parsimonious models for the two long-distance migrants reed warbler and willow warbler. With respect to the latter species our results contrasts with previous findings that rainfall in the Sahel zone does not account for variation in survival rates of reed warblers [65]. Thus, our result is consistent with a number studies that have found that rainfall or its respective surrogates in the Sahel zone is associated with survival or population fluctuations of various migrating passerine species [8], [17], [63], [66]–[68]. Although reed warblers and willow warblers spend the non-breeding season in more southern, moister savannas [69], conditions in the Sahel zone may be crucial for fuel accumulation before the crossing of the Sahara desert during spring migration. In line with our findings is that numbers of barn swallows *Hirundo rustica* breeding in the UK was correlated with rainfall in the western Sahel zone but not with rain fall in the winter quarters further south [70] and similar findings for the nightingale [17].

Unexpected was the high support of the model including the Guinea precipitation index by the data of the blackcap. Some blackcaps do winter in the African Guinea savanna zone [71]. A few band recoveries suggest those to be birds from the British Isles, France, Belgium or Germany [72], but the majority of blackcaps from south-western Germany are expected to winter mostly in the western Mediterranean or north-west Europe [28]. Therefore the result for the blackcap suggests either that blackcaps from the study area spend the winter in hitherto unknown regions in Africa or that our result is an artefact, with the precipitation index from a region far away from the wintering grounds of German blackcaps being associated with an unknown variable important for the apparent survival of blackcaps. The latter is definitely the case for the association of rainfall in the Sahel zone with apparent survival of the dunnock as the species has never been recorded in sub-Saharan Africa [73].

### Single weather factors do not explain a large amount of between-year apparent survival

No single facet of weather explained a high proportion of variance in apparent survival, which indicates the importance of any single aspect of weather on survival should not be over-generalized. Our results are in contrast to the conclusions of a number of previous studies [16], [17], [18], [38], [67]. The reasons for the discrepancy could be that we have selected weather features that do not affect apparent survival to a large extent or that the spatial scale of weather variables as well as the birds' distribution is too large to reveal any association of weather with demography. However, most of the weather variables that we considered have also been associated either with apparent survival or with the population dynamics in previous studies using similar methodologies. However, many previous studies considered only a single weather variable or weather variables during a restricted period within the year. In contrast, studies considering various weather variables experienced throughout the annual cycle also found support for the influence of multiple factors on birds' demography and population dynamics [10], [20], [63], [74].

Our results suggest complex relationships between survival rates of birds and weather, and also indicate spatial and temporal variation in the effects of weather on survival. For migratory birds a significant correlation of the conditions in the wintering areas with apparent survival does not preclude the possibility that conditions experienced on migration or on the breeding grounds will account for a similar or higher proportion of the variation of apparent survival. For example, while survival of white storks was strongly associated with primary production in the African Sahel zone, precipitation during the breeding season also explained some variation of survival [9]. For the same species density on the breeding grounds was associated with precipitation in the Sahel region as well as with winter NAO that affects populations on the breeding grounds [75]. Multiple factors may also affect populations whose members differ in migration routes and wintering areas. In line with this suggestion, models containing the large scale climate variable NAO received strong support for the partial migrant dunnock where individuals from the study site may migrate to different wintering areas [26] and for the long-distance migrant reed warbler.

### Implications for predicting effects of future climate change

The effects of important weather features were in the directions that we expected in most cases. Warmer winters and those with relatively fewer days with snow cover at the study site were associated with higher apparent survival of resident blackbirds as well as of the short-distance migrants dunnock (adults) and chiffchaff. Assuming that this association is due to reduced winter mortality under more benign conditions, this association may indicate a selective advantage under the predicted warmer winters for Central Europe [12] for resident species and individuals that avoid the costs of migration [40]. Analyses showing that blackbirds became less migratory in recent decades [76] and an increasing number of winter observations of chiffchaffs in the vicinity of the study site [30] are consistent with the latter notion. However, although the great majority of chiffchaffs breeding in the study area and all individuals of the long-distance migrant willow warbler spend the winter months away from the study area, apparent survival of these species was associated with the number of days with snow cover on the breeding grounds. For another long-distance migrant warbler of the same genus, the wood warbler, the association of population size with winter severity in Europe has been discussed as being a response to delayed growth of vegetation in spring with a subsequently reduced breeding success [50]. All three *Phylloscopus* warblers breed on the ground and breeding success may strongly depend on adequate nest concealment. Therefore a harsh winter may affect apparent survival of species similarly regardless of their migratoriness, but not necessarily by affecting survival directly via a higher mortality, but instead by inducing dispersal to more suitable breeding sites.

Winter precipitation in the western Mediterranean is expected to decrease [12], [14]. According to our study this may increase the apparent survival probability of the short-distance migrant reed bunting and blackcap (first year birds) as well as the long-distance migrant reed warbler. This finding, as well as the linear increase in apparent survival of the willow warbler, would be counter-intuitive to suggestions that long-distance migrants are most likely to suffer from climate change [77], [78] (but see [79] for reed warbler) and that decreases of willow warbler populations are caused by reduced survival during the non-breeding season [64], [80]. As predicted the Sahel precipitation index is included in the most parsimonious models for the two long-distance migrants, reed warbler and willow warbler. In general it is assumed that true survival of long-distance migrants is negatively affected by droughts in the Sahel region through reduced food supply [16], [66], [74], [80], [81] indicating reduced survival of migrants in times of predicted increasing droughts in this region [14].

Although the directions of weather effects that we identified met our a priori expectations in the majority of cases and there are clear predictions for future climates, we caution against the use of simple associations to predict future fates of populations because: (1) Single climatic factors explained only a small proportion of variation in apparent survival and therefore the remaining variation in survival rates was either random or due to factors outside of the weather features that we and previous studies have suggested to be important determinants of survival. There is always the possibility that important weather factors were missed in our data set, but the inclusion of weather factors acting at different times of the annual cycle of multiple bird species, and correlations among facets of weather (see Methods) suggest it is unlikely that our analyses entirely missed some important features of weather. This leaves a great deal of variation in survival rates that cannot be predicted by simple and direct changes in climate. (2) Birds have responded to the recent climate change by shifting areas of distribution, changes in reproductive phenology and changes in migratory behaviour [82]–[84]. Therefore predictions based on the assumption of a stable association of demography with environmental conditions in a changing world ignore the high potential for phenotypic plasticity or evolutionary adaptation. (3) There may be a trade-off between benefits mediated by climate change in one season against disadvantages in another season, for example when the conditions for reproduction improve on the breeding grounds, but the conditions for survival deteriorate in the wintering areas of long-distance migrants as in our study. The outcome of this trade-off will be difficult to model when the magnitudes of change for several variables and the potentials for response are not known. Finally (4) predicting the future of populations of single species ignores potential interactions between species as community dynamics cannot be inferred from the sum of the predicted behaviours of single species [85] (but see e.g. [86] for a community of moths). Giving these reasons the extrapolation of present conditions to future climate scenarios to explain fates of populations may not be adequate because multiple factors and new climates [87] will create new no-analog communities and different behaviours and adaptations compared to present conditions [88].
